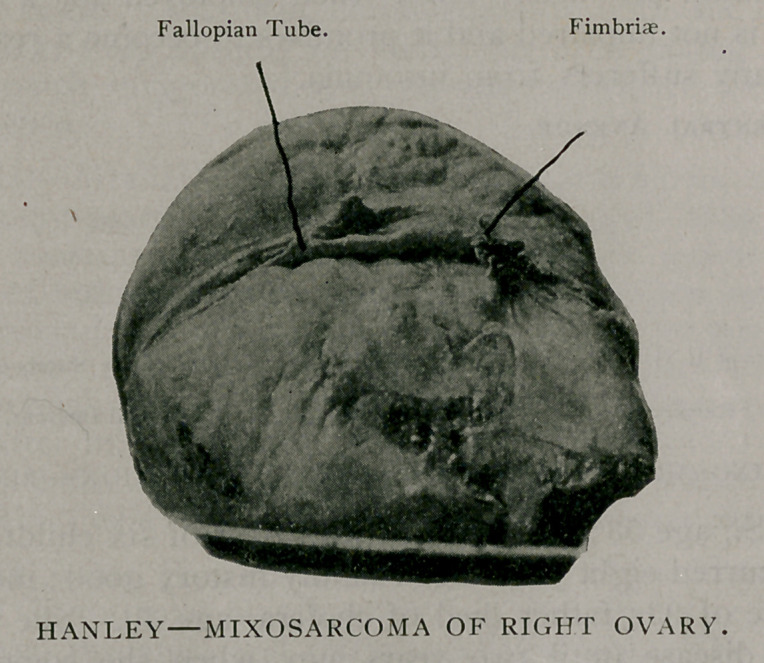# Clinical Report

**Published:** 1903-10

**Authors:** 

**Affiliations:** Hospital Interne; Hospital Interne


### CLINICAL REPORT.

From clinic of L. G. HANLEY, M. D., at the Buffalo Hospital of the Sisters of Charity.

Reported by Drs. Washburn and Highland, Hospital Internes.

### I. MIXOSARCOMA OF RIGHT OVARY. OPERATION—RECOVERY.

Mrs. S., age 53; nativity, Irish; mother of six children, menopause occurred eight years ago; family history good; mother died at the age of 93; father died of cholera, age 30. She has never had any disease until two years ago, when she complained of dragging pain in the abdomen, and noticed, as she says, that she was getting large. She presented herself to hospital for operation.

On examination by palpation, the abdomen seemed to be greatly distended with fluid, and within this cavity was a rather large tumor easily movable. Patient prepared for operation. On opening abdomen two gallons of syrupy, strawcolored fluid were removed. A tumor of right ovary, measuring 11¼ inches long, 8 inches broad and 4 inches deep, weighing 6¼ pounds, was also removed. In attempting to deliver the tumor, it ruptured on its lower border: the intestines, omentum, peritoneum, were covered with metastases. Microscopical examination showed it to be a mixosarcoma of the ovary. Examination made at the Cancer Laboratory, Buffalo University. The tumor presented several areas of hardened tissues; also a gelatinoid condition. The photograph indicates its general appearance. Patient left hospital September 1.

### II. HEMATOCOLPOS CONGENITAL.

Miss K., aged 16; admitted to hospital June 1, 1903, had been complaining for two years; patient anemic, poorly nourished. There was complete occlusion of orificium vaginæ with a marked protrusion of vulva, measuring 4 by 3 inches, which was increased by making pressure over region of uterus. Patient was prepared for operation and an incision 1¼ inches long was made in the direction of the normal opening; the membrane was one-fourth of an inch thick; two quarts of grumous blood, the consistency of melted chocolate, were drawned off from the vagina; there was

HANLEY—MIXOSARCOMA OF RIGHT OVARY.

also hematometra; discharge continued for two weeks. During irrigation it was noticed that the large cavity in the vagina kept gradually contracting until there was complete cessation of discharge. Patient left the hospital three weeks after operation.

### III. APPENDECTOMY. OXYURIS VERMICULARIS IN APPENDIX.

Mrs. S., aged 35; had been ailing for sixteen months and treated, as she says, for one continued attack of appendicitis. Symptoms complained of when admitted to hospital were bloating, pain in right iliac region and at night as though there was a crawling, tingling sensation, which prevented her from sleeping. Patient prepared for operation, though I was not satisfied there were sufficient evidences of appendicitis. Yet, placing a great deal of confidence in my confrère, who directed the case to me, I decided to operate.

The appendix was about 4 inches long, somewhat thickened, with no appearance that there had been any severe inflammatory action. It was removed. On splitting it open it was found to be filled with thread worms—oxyuris vermicularis. Irrigation of bowel, with saline solution, revealed the presence of numbers of this parasite.

I see no reason why this parasite could not invade the appendix as well as the other part of the alimentary tract, but to me it is the first time that I have discovered the presence of this worm in the appendix. It is my custom to open all appendices after their removal, and in upward of 500 cases, this is the first time I have observed such a complication.

## Figures and Tables

**Figure f1:**